# QTL global meta-analysis: are trait determining genes clustered?

**DOI:** 10.1186/1471-2164-10-184

**Published:** 2009-04-24

**Authors:** Hanni Salih, David L Adelson

**Affiliations:** 1 Department of Animal Science and Interdisciplinary Faculty of Genetics, Texas A&M University, 2471 TAMU, Kleberg Center, College Station, TX, USA; 2 School of Molecular and Biomedical Science, The University of Adelaide, 108 Oliphant, Adelaide, SA, Australia

## Abstract

**Background:**

A key open question in biology is if genes are physically clustered with respect to their known functions or phenotypic effects. This is of particular interest for Quantitative Trait Loci (QTL) where a QTL region could contain a number of genes that contribute to the trait being measured.

**Results:**

We observed a significant increase in gene density within QTL regions compared to non-QTL regions and/or the entire bovine genome. By grouping QTL from the Bovine QTL Viewer database into 8 categories of non-redundant regions, we have been able to analyze gene density and gene function distribution, based on Gene Ontology (GO) with relation to their location within QTL regions, outside of QTL regions and across the entire bovine genome. We identified a number of GO terms that were significantly over represented within particular QTL categories. Furthermore, select GO terms expected to be associated with the QTL category based on common biological knowledge have also proved to be significantly over represented in QTL regions.

**Conclusion:**

Our analysis provides evidence of over represented GO terms in QTL regions. This increased GO term density indicates possible clustering of gene functions within QTL regions of the bovine genome. Genes with similar functions may be grouped in specific locales and could be contributing to QTL traits. Moreover, we have identified over-represented GO terminology that from a biological standpoint, makes sense with respect to QTL category type.

## Background

Gene density has been shown to vary widely by organism and genomic region and has been measured both in terms of mean interval between genes and genes per mega base pair of DNA [1,2]. It is known that gene density is positively correlated with G+C content [2] and that the heterochromatic regions surrounding centromeres and telomeres have a lower than average gene density [3-5]. In general, measurements of gene density have focused on correlations of gene density with chromosomal structure or base composition [2,6]. However, to our knowledge no one has looked at the correlation of gene density with Quantitative Trait Locus (QTL) density over the genome. Furthermore, gene density on its own is a fairly crude measurement of the functional role of specific genomic domains. It would be more informative to combine this with quantitative information about the types of gene annotations found across the genome, but to date this has not been done. In this report we describe the correlation of gene density with chromosomal regions defined on the basis of their association with phenotypic traits (QTL regions) and we have determined if gene annotations associated with the phenotypes in question are over represented in these same regions. Our model system is the bovine genome because it has a wealth of well annotated QTL [7] and gene models that have been anchored to a high quality draft genome sequence assembly.

While quantifying gene annotations on the basis of gene descriptions is virtually impossible, quantitative distributions of gene function can be determined on the basis of Gene Ontology (GO) term annotations [8]. A gene ontology is a controlled vocabulary within a structured hierarchy that describes gene products in a species independent manner. For us, GO terms provide a straightforward link from gene coordinates to phenotype. Gene ontologies have been used in many ways for the quantitative analysis of gene expression profiles, for gene set analysis and for general annotation analyses [9-11]. From our perspective, identifying over represented GO terms can provide insight into regional genomic function, and while statistical methods of measuring GO term distribution vary, we have adopted a commonly used method based on the hypergeometric distribution [12].

Until now, GO term analysis performed on the bovine genome has focused on very specific gene expression analysis [13-15]. We have carried out the first genome wide analysis of GO term use correlated with genomic regions known to control quantitatively regulated phenotypes (QTL). One of the challenges of mining GO terms is the large number of GO terms that are often not grouped very tightly by phenotype. One way of overcoming this problem is to use a GO slim, which is a cut down version of the GO. A GO slim contains a subset of terms in the whole GO and facilitates research by streamlining the ontologies for specific areas of interest [8]. At the time we undertook this study there was no bovine GO slim, so we have created our own for this analysis and have deposited it with the GO consortium.

## Results and discussion

### QTL region breakdown

**Figure 1:**
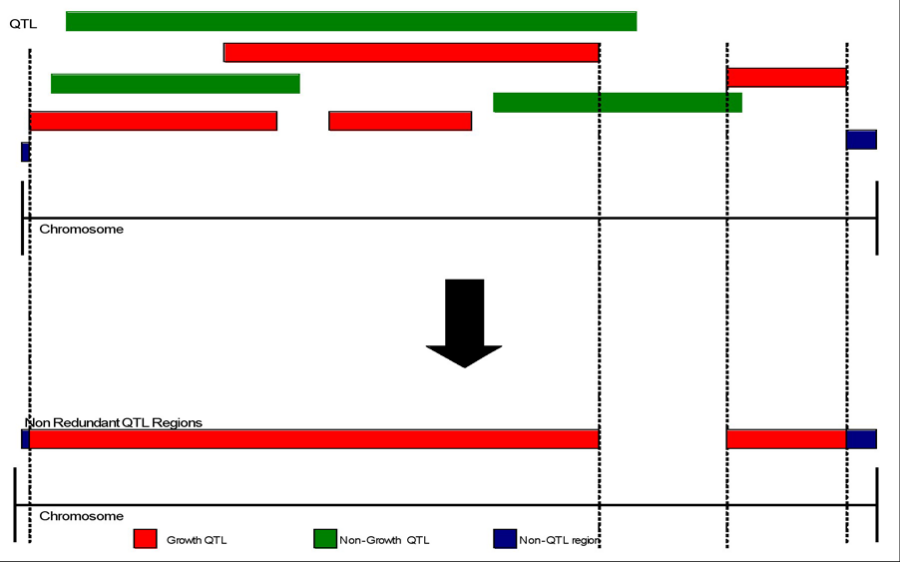
**Grouping QTL into non-redundant regions**. QTL were grouped into 'non-redundant' QTL regions by combining QTL length overlaps into single contiguous regions. The figure illustrates a sample of Growth QTL category QTL being combined to generate a non-redundant region. Non-QTL regions contain no QTL whatsoever.

**Figure 2:**
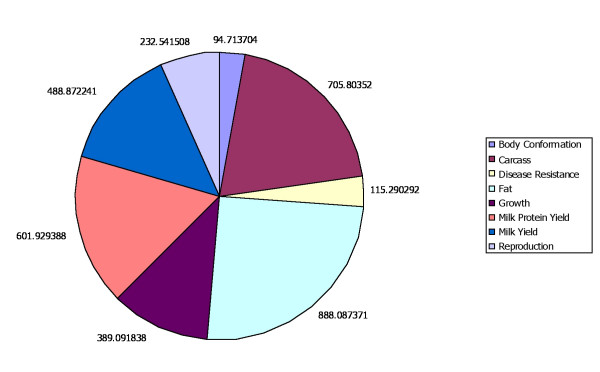
**QTL category size distribution**. Pie chart illustrates the distribution of QTL category sizes. 'Fat' produced the largest non-redundant QTL category of the genome, while 'Body Conformation' produced the smallest. Note that some QTL regions from different categories overlap, leading to a total length of QTL regions that is longer than the genome as a whole.

QTL from the Bovine QTL Viewer database [7] were anchored to *Bos taurus *assembly 3.1 (Btau 3.1) and QTL vs non-QTL (no QTL coverage) regions were identified. Btau 3.1 is approximately 2687 Mb in size, and after QTL placement we have shown that 36.6% of the assembly has not been shown to contain a QTL (non-QTL regions). Mapped QTL lie within the remaining 63.4% of the genome, and these regions were classified to create 8 distinctive phenotypic categories of QTL. QTL within individual categories were collapsed on the basis of overlap for the purpose of identifying non-redundant regions (Fig. 1) of the genome associated with particular phenotypes. For example, the QTL category for adiposity "Fat" includes the greatest number of base pairs of the non-redundant QTL regions (52.1%) and is composed of the highest number of QTL; 161. Conversely the "Body Conformation" QTL category spans the smallest number of base pairs of the non-redundant QTL regions (5.5%), and the "Disease Resistance" QTL category has the fewest number of contributing QTL; 25 (Fig. 2).

### Gene density analysis

Partitioning the QTL into non-redundant classes provided the opportunity to quantify correlations between gene density and their associated ontology term frequencies. We measured the distance between the probability distributions for gene density between QTL and non-QTL regions using Welch's t-test. The advantage of this method over using the raw gene count distribution is that this approach gives very little weight to the 0 or low gene counts which would otherwise distort the result. Our analysis (see Additional File 1) demonstrated a statistically significant enrichment of gene density in QTL regions (average of 5.3 genes/5 Mbp bin) compared to non-QTL regions (average of 2.3 genes/5 Mbp bin) (p-value 0.042). This could indicate that genes are clustered in regions of the bovine genome that contribute to quantitative traits. We were not convinced that this correlation of increased gene density with QTL regions indicated functional clustering of genes in those regions. For this reason we decided to examine the quantitative distributions of gene functions with respect to QTL regions.

### Enriched term (GO) t-test analysis

To measure the distribution of genes according to function with respect to QTL regions, we analyzed GO term frequencies with the aim of identifying significantly enriched terms. We did this by searching for over-represented terms in each QTL category, *i.e. *significantly enriched terms when compared to the full genome. We performed this analysis using two different methods. In our first approach we binned the genome into 5 Mb regions, and then counted the number of times each GO term occurred and normalized that with respect to gene density. The resulting distributions of GO term frequency were normalized using a log transformation and then Welch's t-test was used to determine significance. Because this would have resulted in an enormous number of tests, each with low counts, we cycled all GO terms up to their second level and searched for over representation of those terms in the 8 QTL regions, after applying a Bonferroni correction to take into account multiple testing. The GO enrichment t-test analysis showed a single term to be over represented with respect to genome wide occurrence (Table 1). The term 'binding' (GO: 0005488), showed significant enrichment during t-test analysis (0.00765% of total GO terms analyzed, 3% of GO terms analyzed within 'Reproduction' QTL category). Binding refers to the interaction of a molecule with a specific site on another molecule. The term 'binding' could plausibly be involved in processes of the overall QTL category 'Reproduction' where it was enriched. We found two terms whose frequency was lower in Reproduction QTL regions; 'synapse' (GO: 0045202) and 'synapse part' (GO:0044456). Genes annotated with 'synapse' and 'synapse part' do not have an obvious relationship with 'Reproduction'. Because the analytical approach we used was novel, we elected to use a well-known method to confirm our analysis.

**Table 1:** Second level GO terms that differed when comparing QTL to the full genome using the t-test.

**QTL Category**	**GO Term**	**Description**	**QTL GO term density**	**Genome wide GO term density**	**Ratio**	**p. value**
Reproduction	GO:0005488	binding	1.00	0.83	1.19	0.0001
Reproduction	GO:0045202	synapse	0.03	0.05	0.64	0.0032
Reproduction	GO:0044456	synapse part	0.03	0.04	0.65	0.0053

Table describes second level GO terms found to be significantly different via t-test analysis. GO term densities are mean values normalized with respect to gene content. To be considered statistically significant, the p-value must be less than 0.00625 (Bonferroni corrected). QTL category describes the QTL group from which the terms where found in abundance.

### Enriched term (GO) GeneMerge analysis

To confirm the significant over represented GO terminology across QTL regions/categories observed above, we used the GeneMerge software tool [16], based on a hypergeometric distribution. Note that this method does not take into account differences in gene density. Because our dataset was moderate in size, a hypergeometric distribution analysis was suitable [17,18]. In addition, GeneMerge allows for selection of population and sub-population genes allowing us to compare QTL (sub-population) genes to the full genome genes (population). By using the second level GO terms as the subpopulation, we identified a number of terms found that were significantly over represented (Table 2). A total of 22 of 49 second level GO terms (44%) were found to be significantly over represented in their respective QTL categories. Most notably the term 'binding' (GO: 0008152) showed significant over representation corroborating the result of the t-test analysis. This provided a strong indication that gene products involved in 'binding' were clustered in QTL regions associated with reproduction phenotypes. Other over represented terms had a clearer biological association with the relevant QTL category such as: 'transporter activity' (GO:0005215) enriched in the 'Growth' QTL category or 'transcription regulator activity' (GO:0030528) enriched in the 'Milk Yield' category.

**Table 2:** Second level GO terms that differed when comparing QTL to the entire genome using GeneMerge.

**QTL Category**	**GO Code**	**Description**	**Population Frequency**	**QTL/Population**	**p value**
Body Conformation	GO:0044456	synapse part	0.00429	4.00155	0.0004
Body Conformation	GO:0045202	synapse	0.00810	3.06373	0.0004
Disease Resistance	GO:0022414	reproductive process	0.00664	2.31530	0.0030
Fat	GO:0040007	growth	0.01040	1.20290	0.0197
Growth	GO:0005623	cell	0.42688	1.05397	0.0014
Growth	GO:0031974	membrane-enclosed lumen	0.02531	1.24800	0.0070
Growth	GO:0044464	cell part	0.42683	1.05408	0.0014
Growth	GO:0009987	cellular process	0.40776	1.06011	0.0007
Growth	GO:0005215	transporter activity	0.05160	1.18584	0.0034
Growth	GO:0030528	transcription regulator activity	0.04947	1.15047	0.0161
Milk Protein	GO:0044421	extracellular region part	0.01633	1.21375	0.0196
Milk Yield	GO:0005623	cell	0.42688	1.04582	0.0041
Milk Yield	GO:0044464	cell part	0.42683	1.04531	0.0044
Milk Yield	GO:0009987	cellular process	0.40776	1.04050	0.0123
Milk Yield	GO:0030528	transcription regulator activity	0.04947	1.16269	0.0077
Milk Yield	GO:0043234	protein complex	0.07580	1.11389	0.0160
Reproduction	GO:0008152	metabolic process	0.27284	1.05689	0.0174
Reproduction	GO:0005488	binding	0.38440	1.06651	0.0007
Reproduction	GO:0009987	cellular process	0.40776	1.04258	0.0159
Reproduction	GO:0030528	transcription regulator activity	0.04947	1.24460	0.0005
Reproduction	GO:0043226	organelle	0.25103	1.08465	0.0015
Reproduction	GO:0005198	structural molecule activity	0.02566	1.26584	0.0059

The table describes second level GO terms found to be significantly over represented via hypergeometric analysis. The 'QTL categories' group provides the category from which the subsequent terms were found to be in abundance. Population frequency describes the frequency of the occurrence of the term genome wide. QTL/Population describes the ratio of the frequency of a GO code within a QTL region compared to the frequency of that GO code genome wide.

**Figure 3:**
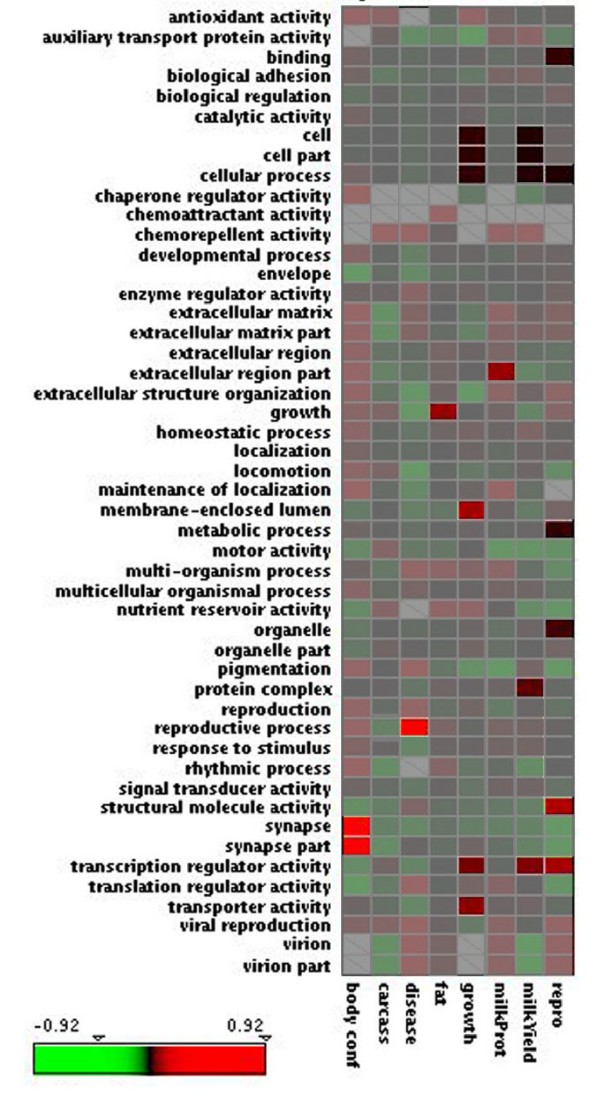
**GeneMerge analysis heat map**. Each box represents the ratio of GO term frequency in the QTL category to the full genome [25]. In order to better visualize the differences, the frequencies were log transformed to generate the heat map. Red indicates a higher frequency in the QTL regions, green indicates a higher frequency across the genome. White boxes represent GO terms that were not found in the QTL category regions. Some GO terms from specific categories have shown statistical significant over representation when compared to the full genome. The grayed areas of the figure are not statistically significant, while the vibrant colors highlight the statistically significant GO term differences.

In order to visualize the magnitude of the GO term enrichment, we calculated the ratio of the GO frequencies in QTL regions to the full genome frequencies (Fig. 3). The more frequent a QTL region GO term was relative to the full genome, the redder the box. Conversely, the less frequent a GO term was relative to the full genome, the greener the box. Frequency ratios that were not significantly different were greyed out on the heat map. Terms whose frequencies were found to differ significantly showed a tendency to be over, rather than under represented. Some significant terms such as 'synapse' (GO:0045202) and 'synapse part' (GO:0044456) and 'reproductive process' (GO:022414) were at very high frequency within their respective QTL regions compared to the genome as a whole. In these cases, the QTL regions were the smallest, possibly indicating that gene clusters/families in these small regions could be driving the frequency ratios up. Most other GO terms in Table 2 showed a frequency ratio very close to 1.0 while still showing statistically significant over representation (i.e. 'Growth'-'Cell' (GO:0005620), 'Growth'-'Cell Part'(GO:0044464)). The association of the 'Reproduction' QTL regions and the 'binding' GO term category was found when comparing QTL to non-QTL regions, or the genome as a whole. These second level GO terms allowed us to compare low granularity QTL regions to low granularity GO terms, possibly exposing associations of broad biological functions with broadly grouped phenotypes. This type of analysis trades off specifically meaningful annotations in favor of sometimes rather uninformative ones. In some cases high level GO terms showed no correlation with QTL categories whose meanings were similar, such as 'reproductive process' and 'reproduction' GO terms with the 'reproduction' QTL category. In order to address this, we moved our comparisons further along the ontology to provide a higher level of specificity.

Lowest level GO terms were analyzed for over representation within QTL regions using GeneMerge (Table 3). As a result, 45 GO terms (0.00025%) were found to be significantly more prevalent within QTL regions. Again, the two QTL categories encompassing the smallest number of base pairs had the highest number of correlated GO terms, possibly indicating a sample size effect. When comparing the GO terms across QTL categories, it was obvious that there was some overlap of GO terms across QTL regions. This was probably a function of physically overlapping QTL regions resulting from traits that are known to be correlated such as the 'fat', 'carcass' and 'milk protein' traits. In this case, the two GO terms found for 'fat' were included in the other two QTL categories. It is known that milk fat content is inversely correlated with milk protein content [19] and that intramuscular fat accumulation is positively correlated with carcass traits that measure meat tenderness [20]. Of all the QTL categories, milk yield showed the most interesting, or 'biologically plausible' set of associated GO terms. Most of the time, however, there was not a clear relationship between GO terms and the associated QTL category. This could be because many GO terms are not clearly associated with specific phenotypes, in spite of being useful biological annotations. Or, it could reflect our sketchy understanding of the genetic networks that underlie many phenotypes.

**Table 3:** Lowest level GO terms that differed in QTL regions compared to the genome as a whole.

**GO Term**	**Description**	**Population Frequency**	**QTL/Population**	**p-value**
**Body Conformation**				
GO:0005254	chloride channel activity	0.00115	8.29	2.89E-04
GO:0030594	neurotransmitter receptor activity	0.00159	8.39	1.61E-05
GO:0048500	signal recognition particle	0.00040	14.38	9.38E-04
GO:0004890	GABA-A receptor activity	0.00102	11.25	1.09E-05
GO:0005230	extracellular ligand-gated ion channel activity	0.00159	9.58	1.35E-06
GO:0007214	gamma-aminobutyric acid signaling pathway	0.00111	8.63	2.38E-04
GO:0045202	synapse	0.00766	2.99	7.42E-04
GO:0045211	postsynaptic membrane	0.00394	4.36	2.22E-04
**Carcass**				
GO:0050806	positive regulation of synaptic transmission	0.00031	3.38	2.00E-04
GO:0016600	flotillin complex	0.00031	3.38	2.00E-04
GO:0051059	NF-kappaB binding	0.00088	2.36	2.24E-04
GO:0006986	response to unfolded protein	0.00208	1.87	2.07E-04
**Disease Resistance**				
GO:0046785	microtubule polymerization	0.00035	15.50	5.33E-06
GO:0031116	positive regulation of microtubule polymerization	0.00035	15.50	5.33E-06
GO:0009925	basal plasma membrane	0.00049	13.53	1.64E-06
GO:0001937	negative regulation of endothelial cell proliferation	0.00044	12.40	2.24E-05
GO:0007154	cell communication	0.00389	3.38	2.10E-04
GO:0042493	response to drug	0.00195	4.51	3.30E-04
GO:0004620	phospholipase activity	0.00022	19.85	1.27E-05
GO:0019900	kinase binding	0.00058	9.54	1.03E-04
GO:0042470	melanosome	0.00049	9.02	6.90E-04
GO:0043434	response to peptide hormone stimulus	0.00084	6.53	7.63E-04
GO:0030659	cytoplasmic vesicle membrane	0.00035	12.40	1.61E-04
GO:0016599	caveola	0.00053	10.34	6.58E-05
GO:0019905	syntaxin binding	0.00058	9.54	1.03E-04
GO:0009395	phospholipid catabolic process	0.00031	14.18	8.33E-05
GO:0050998	nitric-oxide synthase binding	0.00035	15.50	5.33E-06
GO:0030321	transepithelial chloride transport	0.00031	14.18	8.33E-05
GO:0042311	vasodilation	0.00040	11.03	2.81E-04
GO:0019861	flagellum	0.00111	5.95	3.87E-04
GO:0007595	lactation	0.00084	6.53	7.63E-04
GO:0030317	sperm motility	0.00084	7.83	7.29E-05
**Fat**				
GO:0051059	NF-kappaB binding	0.00088	2.33	1.06E-04
GO:0006986	response to unfolded protein	0.00208	1.85	1.01E-04
**Growth**				
GO:0007586	digestion	0.00133	2.79	2.66E-04
**Milk Protein**				
GO:0050785	advanced glycation end-product receptor activity	0.00027	4.67	9.67E-05
GO:0051059	NF-kappaB binding	0.00088	3.27	4.36E-06
GO:0006986	response to unfolded protein	0.00208	2.28	2.71E-05
GO:0007584	response to nutrient	0.00217	2.19	6.25E-05
**Milk Yield**				
GO:0005922	connexon complex	0.00080	3.31	2.05E-04
GO:0030375	thyroid hormone receptor coactivator activity	0.00040	4.64	9.74E-05
GO:0042809	vitamin D receptor binding	0.00071	3.35	3.82E-04
GO:0042974	retinoic acid receptor binding	0.00031	5.96	3.71E-06
GO:0004886	retinoid-X receptor activity	0.00044	4.77	2.03E-05
**Reproduction**				
GO:0005882	intermediate filament	0.00186	3.16	1.38E-06

The table describes fine detail GO terms found to be significantly over represented through hypergeometric analysis. In bold are the QTL categories from which the subsequent terms were found to be in abundance. Population frequency describes the frequency of the occurrence of the GO code genome wide. QTL/Population describes the ratio of the frequency of a GO code within a QTL region compared to the frequency of that GO code genome wide.

This type of analysis is biased by the nature and comprehensiveness of the annotations in the Gene Ontology and by the number of GO annotated gene models in the bovine genome. It is beyond the scope of this report to comment on the former, but since the bovine annotations depend overwhelmingly on the transfer of GO annotations from human, we know that many of the bovine gene models remain un-annotated. It is also likely that in spite of stringent sequence similarity criteria for the transfer of GO annotations that some will be incorrect.

### GO slim result

During the course of this analysis a GO slim was created to reduce the large number of total GO terms to create a list of terms more specific for bovine analyses. We identified 272 terms whose meanings associated them with QTL, or terms enriched in QTL regions, or terms commonly known to be associated with physiologically/commercially important bovine traits that did not correspond to QTL.

## Conclusion

The idea that genes are not randomly distributed throughout the genome can be traced back to R.A. Fisher [21], who showed that interacting genes tend to become more closely linked. More recently, tissue specific patterns of gene expression have been shown to map to chromosomal domains [22]. Our quantitative analysis of the gene content of QTL regions should be viewed in this context, and was able to provide evidence that gene density is higher in QTL regions and that some gene functions, as reflected by GO terms are also over-represented in QTL regions. While many of the GO terms found to be associated with QTL categories were not obviously linked through a biological context, these results were consistent with the hypothesis that genes may be clustered in a manner that reflects their functional association with particular traits. This was most obvious for the 'milk yield' QTL category, where the associated GO terms were highly biologically plausible.

## Methods

### Placement of additional USDA Marc Markers onto Btau 3.1 assembly through BLAST and E-PCR

STS sequence data was downloaded from the NCBI website. All markers with unknown locations (*i.e. *not in the STS data from NCBI) were aligned to the Btau 3.1 assembly using MegaBLAST and BLASTN. Markers whose alignments were 100% identical with over 90% of their length were verified by ensuring that each marker could be placed on the same chromosome in both the linkage map and the sequence assembly. This method placed 78 sequence tagged sites onto the assembly. However, after both BLAST analyses, some markers could not be placed on the assembly because they either had no BLAST hit or none of their hits fulfilled the above criteria. There were also a number of linkage markers without NCBI accessions which not be anchored to the genome via BLAST/MegaBLAST due to a lack of sequence data [23]. We attempted to place these markers using e-PCR, permitting 1 gap and 1 mismatch [24]. E-PCR allowed us to place an additional 42 sequence tagged sites on the assembly.

### Gene dataset

14,354 bovine genes were annotated with Gene Ontology data from human orthologs by David Lynn. Additional non-annotated genes from the GLEAN 5 dataset were included for a total of 22,418 bovine gene models. We identified all the bovine gene models within each non-redundant QTL region.

### Non-redundant QTL categories

Each QTL category was collapsed into non-redundant regions. Overlapping QTL regions for each QTL category were combined into single, contiguous non-redundant regions. Figure 1 provides an illustration of how Growth QTL were combined to create non-redundant regions. Non-QTL regions are locations of the genome in which no QTL are known to be present. There were a total of 597 QTL used in this study with the following breakdown: Body Conformation 47, Carcass 94, Disease Resistance 25, Fat 162, Growth 67, Milk Protein Yield 114, Milk Yield 61 and Reproduction 35.

### Binning strategy employed for t-tests

QTL category regions and non-QTL regions of the genome were divided into sequential 5 Mb "bins." Gene counts/GO term counts were measured in each bin across the regions. For gene counts, a histogram plot of the bin counts showed that the distribution was not normal; mostly due to a large number of zero count bins. For gene density comparison, we transformed the bin counts into probability distributions of gene density, which removed the zero count bins and normalized the distribution. For GO terms similar problems were encountered, with many zero count bins. For this analysis we used a log transformation to remove the zero counts and normalize the distributions (see below).

### GO analysis using counts normalized for gene density

Second level gene ontologies were counted in non-redundant QTL category regions. The structure of GO is that the child terms are to be more specific and targeted than their parent terms. Gene products associated with a GO term are expected to be loosely associated with the parent term and even more loosely associated each term up the ontology. Using a mysql database of GO terms downloaded from the Gene Ontology website  we were able to cycle up terms from associated gene products, to higher parent terms. GO terms can be traversed to multiple parents. So as not to negate any possible contributing factors, all second level GO term parents of child GO terms were counted and incorporated into bin counts.

All the genes within the boundaries of non-redundant QTL regions in each category were identified and the GO terms for each gene were counted. A number of bins produce zero GO terms if: (a) there were no genes present or (b) the genes did not produce terms for specific second level GO. To overcome this problem, we started all counts at 1 rather than zero. The number of genes in a given bin can be a source of ascertainment bias because bins with high numbers of genes will produce high GO counts. In order to control for this, the GO count per bin was divided by the number of genes in the bin. Since the resulting distributions were very skewed, we normalized these by log transformation. Bin values were therefore calculated as follows:



Where *n *= normalized bin value, *x *= gene count, *y *= GO term count

### GeneMerge analysis

GeneMerge analysis was performed according to [16], using the raw GO term counts from all bins across QTL regions and the full genome. Genes within each of the 8 QTL categories were grouped and GO term frequencies associated with gene products from those genes were compared against GO term frequencies found across the genome as a whole (population). By using the second level GO terms from each QTL category regions as the subpopulation, via GeneMerge we identified second level GO terms found to be statistically significantly over represented. We compared fine level GO terms in the same manner; by grouping QTL region GO terms (subpopulation) and comparing against second level GO terms from the entire genome (population). To illustrate the results of the GeneMerge analysis integrated with the ratio of the frequency of GO terms within QTL regions to the genome, the Mayday platform [25] was used to create heat maps. A data file was produced and loaded into Mayday containing QTL categories, GO terms and values of GO term ratios between QTL regions and the genome. Non-statistically significant relationships were washed out by colour shading. The enhanced heat map (Fig. 3) displays which GO terms were significantly enriched, and the extent to which GO term frequency from particular QTL regions vary with respect to the entire genome.

## Authors' contributions

HS produced QTL regions, created scripts to determine GO counts, bin ranges and GO term cycling. DLA helped analyze t-test data and designed the overall experiment. Both authors contributed to manuscript preparation and have read/approved the final manuscript.

## Supplementary Material

Additional file 1 **File contains gene frequency and probability distributions for QTL and non-QTL regions, along with t-test results based on the probability distributions.** Click here for file
