# Supplementary material for: QTL global meta-analysis: are trait determining genes clustered?
Source: BMC Genomics. 2009 Apr 24;10:184. doi: 10.1186/1471-2164-10-184 (PMC2683869; doi:10.1186/1471-2164-10-184)
Supplement: Additional file 1 — File contains gene frequency and probability distributions for QTL and non-QTL regions, along with t-test results based on the probability distributions. [file 1471-2164-10-184-S1.doc]

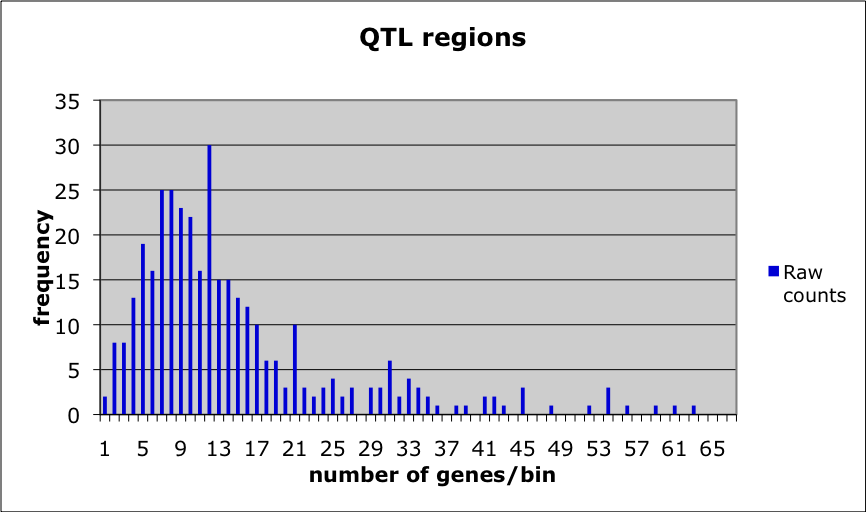


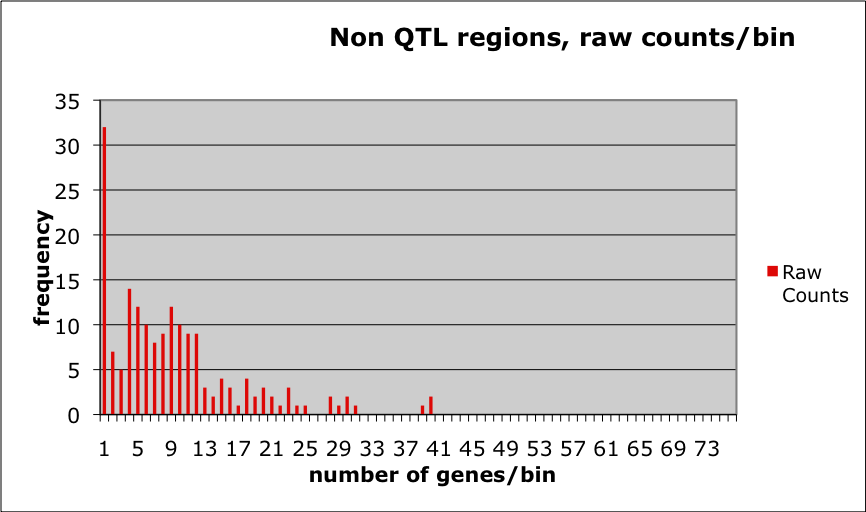


These histograms show the raw counts of genes/5 Mbp bin. Note the large number of occurrences of 0 counts in the Non-QTL regions. These 0 count bins distort the distribution.


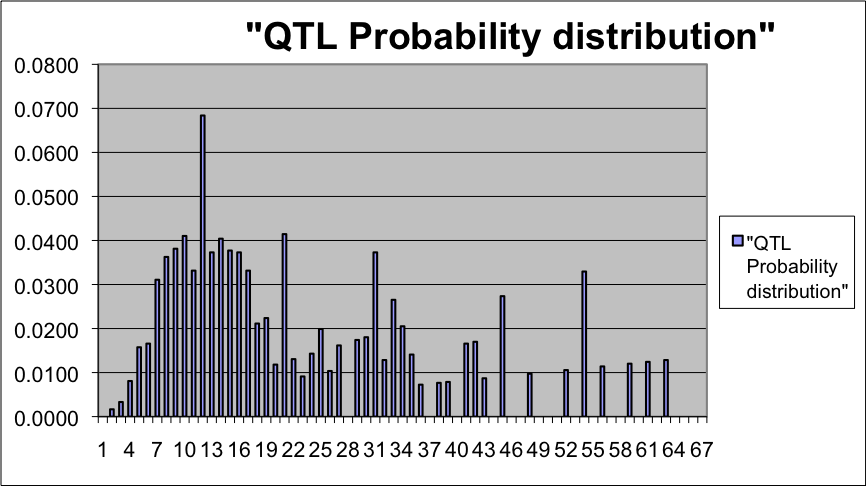


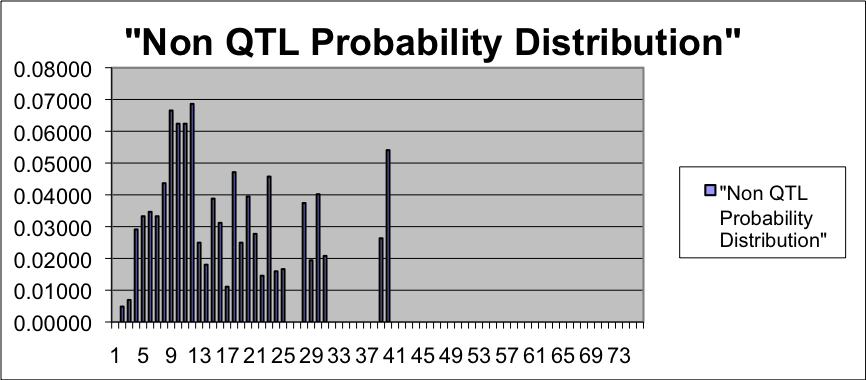


These are the probability distributions of the frequency distributions shown on the previous page. The probability distribution values for each bin are generated by multiplying the bin frequency by the bin size. This removes the large number of zero value bins in the non-QTL regions that would distort the analysis. We report the two tail P value in the main body of the paper.

| t-Test: Two-Sample Assuming Unequal Variances | |  |
| --- | --- | --- |
|  |  |  |
|  | *QTL regions* | *Non QTL regions* |
| Mean prob value | 0.676113836 | 0.426213663 |
| Variance | 0.492229863 | 0.565714705 |
| Observations | 67 | 76 |
| Hypothesized Mean Difference | 0 |  |
| df | 141 |  |
| t Stat | 2.054838247 |  |
| P(T<=t) one-tail | 0.020870403 |  |
| t Critical one-tail | 1.655732288 |  |
| P(T<=t) two-tail | 0.041740806 |  |
| t Critical two-tail | 1.976931458 |  |
|  |  |  |
| This test measures the distance between two probability distributions. The advantage over using the gene count distribution is that this approach gives very little weight to the 0 or low gene counts which would otherwise distort the result. This is not a perfect correction for the biased sampling, but it helps. |  |  |
